# The multi‐factorial modes of action of urease in the pathogenesis of incontinence associated dermatitis

**DOI:** 10.1002/ski2.349

**Published:** 2024-03-02

**Authors:** Emily J. Owen, Rachel A. Heylen, Kyle Stewart, Paul G. Winyard, Andrew Toby A. Jenkins

**Affiliations:** ^1^ Department of Chemistry University of Bath Bath UK; ^2^ Watercress Research Ltd. Exeter UK

## Abstract

**Background:**

Incontinence Associated Dermatitis (IAD) is a type of skin inflammation caused by chronic exposure to urine and/or faeces. Current treatment strategies involve creating a barrier between the skin and urine/faeces rather than targeting specific irritants. Urease expressing pathogens catalyse the conversion of urea, present in urine, into ammonia. The accumulation of ammonia causes an elevation in skin pH which is believed to activate faecal enzymes which damage skin, and opportunistic pathogens, which lead to secondary infections.

**Objectives:**

To develop a better, multi‐factorial model of IAD pathogenesis, including the effect of urease‐expressing bacteria on skin, mechanism of damage of urease and urease‐triggered activity of faecal enzymes and secondary pathogens. To study the effect of urease inhibition on preventing IAD skin damage.

**Methods:**

Five separate studies were made using ex vivo porcine skin and in vivo human skin models. Measurements of the change in skin barrier function were made using skin impedance, trans‐epidermal water loss (TEWL), stratum corneum moisture and pH. Skin was exposed to artificial urine, inoculated with various microbes, enzymes and chemicals to examine the influence of: 1) urease‐positive *Proteus mirabilis* 2) ammonia, 3) combination of *P. mirabilis* and a faecal enzyme, trypsin, 4) combination of *P. mirabilis* and opportunistic pathogens, *Candida albicans* and *Staphylococcus aureus*, 5) inhibition of urease using acetohydroxamic acid (AHA) on barrier function.

**Results:**

The urease‐mediated production of ammonia had two principal effects: it elevated skin pH and caused inflammation, leading to significant breakdown in skin (stratum corneum) barrier function. Urease was found to further increase the activity of faecal enzymes and opportunistic pathogens, due to elevated skin pH. The urease inhibitor, AHA, was shown to have significantly reduced damage to skin barrier function, measured as its electrical resistance.

**Conclusions:**

Targeted therapeutic strategies should be developed to prevent the manifestation of IAD, rather than creating a generic barrier between skin and urine/faeces. Urease has been identified as a crucial component in the manifestation of IAD, due to its role in the production of ammonia. Urease inhibition provides a promising therapeutic target to halt the progression of IAD.

## Graphical abstract

Incontinence associated dermatitis involves the complex interplay of several bacteria, yeast, and enzymes. This paper looked at the various components and their effect on stratum corneum barrier function and the potential role of inhibiting the urease enzyme as a treatment approach.

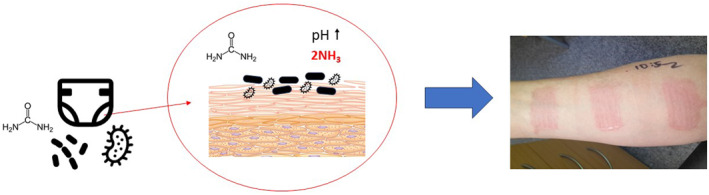

**What's already known about this topic?**

- Incontinence Associated Dermatitis (IAD) is skin inflammation caused by prolonged exposure to urine and/or faeces. Although IAD is usually not life‐threatening, secondary infections can arise which are particularly serious in individuals with comorbidities. Several enzymes, including urease and trypsin, and faecal pathogens are involved in the manifestation of IAD. Current treatment strategies involve creating a barrier between skin and urine/faeces or remove the urine/faeces, to allow skin to heal.

**What does this study add?**

- An improved multi‐factorial model of IAD pathogenesis is proposed with the action of the enzyme urease at its core. Urease‐mediated production of ammonia is a crucial stage. Firstly, ammonia directly damages skin causing a chemical burn. Secondly, the resulting elevated skin pH enhances growth of opportunistic pathogens, morphological changes in *Candida albicans* and elevated protease activity. We present evidence that inhibiting urease is a potentially effective strategy in managing IAD.

**What is the translational message?**

- IAD management strategies are currently focused on use of barrier creams, more frequent pad changes and in babies, where practicable, nappy/diaper free time. The underlying biochemical cause of IAD is not addressed. We suggest that the causative enzyme, urease is a good pharmaceutical target for inhibition which would directly treat IAD. A few urease inhibitors currently exist, we suggest that topical ointments containing such inhibitors might be effective.

## 1 INTRODUCTION

Incontinence Associate Dermatitis (IAD) refers to skin inflammation resulting from prolonged exposure to urine and/or faeces.^1^, ^2^ IAD most commonly affects paediatric and geriatric populations due to these demographics being most prone to incontinence, as well as their skin being more fragile, compared to typical adults.^3^, ^4^ Estimates suggest that IAD has a prevalence of 5.6%–50% and incidence of 3.4%–25% in the non‐infant population.^2^, ^4^, ^5^ Symptoms include pain, pruritus and burning, all of which reduce an individual's quality of life.^5^, ^6^ In severe cases, IAD can lead to secondary infections which can be life‐threatening to frail, immunocompromised individuals or those with complex co‐morbidities.^7^, ^8^ Furthermore, IAD is a known risk factor for development of pressure ulcers: by improving IAD prevention strategies, this may also have a positive impact on pressure ulcer care.^7^ In children and babies, IAD (“nappy/diaper rash”) is the most common dermatological disorder: virtually every child will experience at least one episode.^9^, ^10^, ^11^, ^12^, ^13^ Nappy rash may seem a trivial condition but severe nappy rash may be an indication of child neglect/abuse and has the potential to progress to serious complications which require hospitalisation for example, vulvovaginitis and candidiasis.^9^, ^14^, ^15^

**FIGURE 1:**
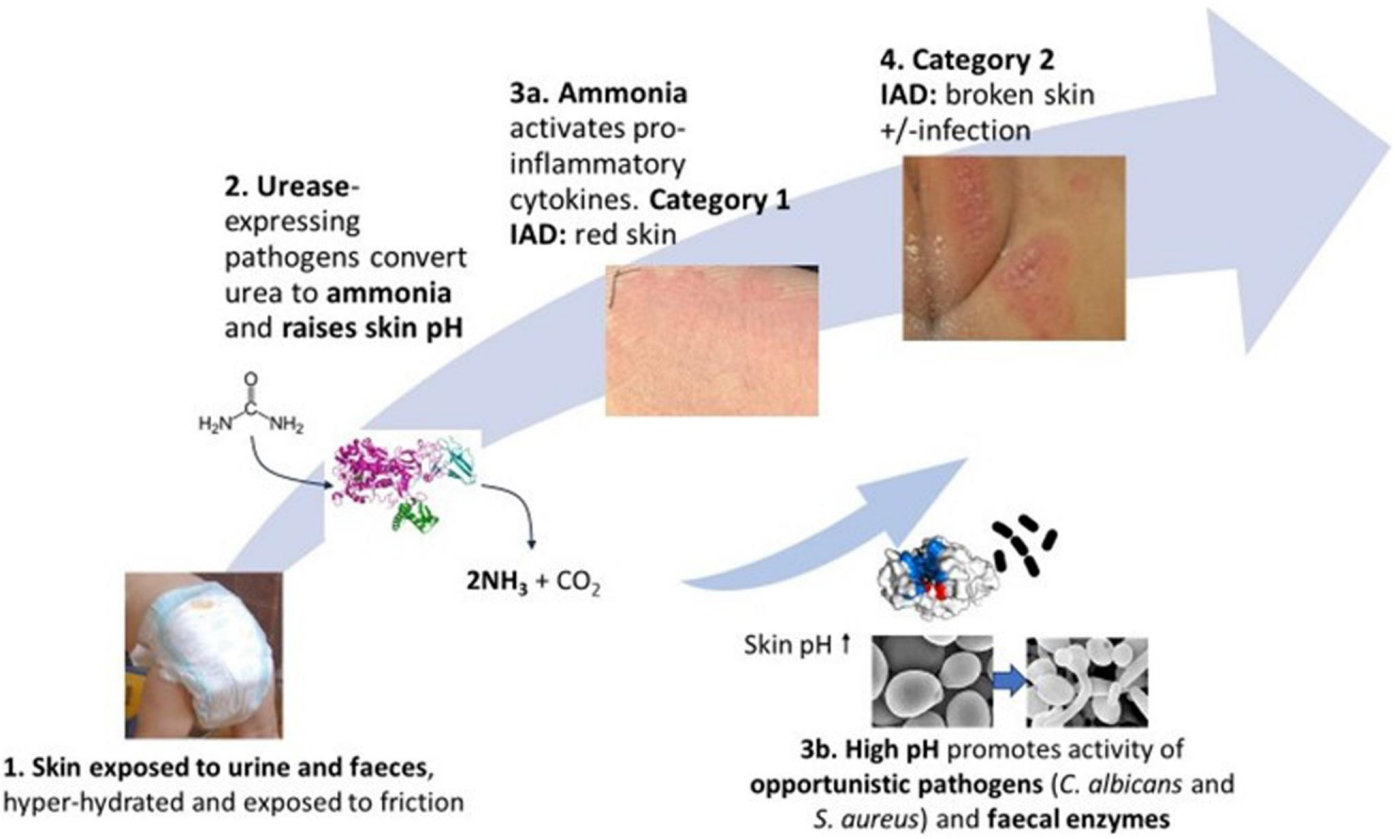
Outline schematic showing the complex multi‐factorial aspects of IAD/nappy rash pathogenesis with urease being central to the process. Ammonia produced by urease directly damages skin, leading to Category 1 IAD, and promotes pathway 3b, leading to Category 2 IAD.

Several key factors have been identified in the aetiology and pathophysiology of IAD (Figure 1), which are studied in this paper.^16^ Currently, there is no internationally accepted method used to diagnose and categorise the severity of IAD.^2^, ^4^, ^5^, ^17^ In this work, IAD was categorised using the Ghent Global IAD Categorisation Tool (GLOBIAD) where persistent redness/erythema is Category 1 and skin loss is Category 2, each presenting with/out clinical signs of infection.^18^ In order to improve diagnosis and management of IAD, particularly in preventing complications arising from severe (Category 2) cases, it may be useful in the future to have techniques such as skin impedance measurement, which give an objective measure of stratum corneum barrier function, rather than relying on visual appearance alone.^1^, ^4^, ^5^, ^18^, ^19^

Current IAD intervention strategies involve removing urine and/or faeces from the affected site, cleansing the skin and applying a skin barrier cream.^4^, ^5^, ^20^ However, preventing further exposure of the skin to urine/faeces poses a significant challenge for incontinent people who use pads and for infants is often impractical. It has been suggested in the literature that chronic exposure to urinary/faecal irritants causes epidermal keratinocytes to release growth factors and pro‐inflammatory cytokines, including IL‐1α, IL‐8 and TNF‐α.^4^, ^21^, ^22^ The process is complex, initially prolonged exposure of skin to urine and/or faeces creates a moist environment. With time, skin maceration can occur which involves hyperhydration of keratinocytes and disruption of intercellular lipid bilayers.^2^, ^5^, ^6^ This results in the skin becoming more susceptible to mechanical damage caused by frictional forces.^4^, ^5^, ^23^ Furthermore, lipolytic and proteolytic enzymes present in faeces, such as trypsin, *α*‐chymotrypsin and lipase, can contribute to tissue damage and skin breakdown.^2^, ^6^, ^24^, ^25^

Certain faecal pathogens including the Gram‐negative bacteria *Proteus mirabilis (P. mirabilis)*, express the enzyme urease.^24^, ^26^, ^27^, ^28^ It has also been reported that skin commensal bacteria including Gram positive *Staphylococcus aureus* (*S. aureus*) express urease, with a recent study examining the role of urease expressing *S. aureus* in persistent urinary tract infections.^29^ Urease catalyses the conversion of urea (present in urine) into ammonia.^19^, ^26^, ^30^ The production of ammonia has two principal effects on skin; increase in skin pH and direct chemical damage leading to erythema. The elevated skin pH is frequently commented on in literature, though the precise effect not always discussed.^16^ For example, commensal skin microbes, such as *Lactobacilli*, are inhibited by raised skin pH allowing opportunistic pathogenic microbes to colonise the skin.^30^, ^31^, ^32^ A 2023 study of incontinent adult patients with IAD by Kota et al showed a significant correlation between urease expressing bacterial on the skin of the patients and IAD incidence.^33^ The most frequently associated pathogens in IAD infections include *Candida albicans* (*C. albicans*) from the gastrointestinal tract, and *S. aureus*, from the perineal skin^2^, ^19^, ^34^ The elevated pH (from ammonia) also enhances the activity of faecal enzymes including proteases and lipases.^4^, ^19^, ^26^, ^36^ Ammonia is a caustic chemical which at sufficiently high concentrations, can cause chemical burns.^16^, ^28^, ^37^

In this paper, we propose an improved multi‐factorial model of IAD formation, examining the effect of urease‐expressing bacteria on skin and urease's mechanism of damage, including direct and indirect consequences of ammonia production. Key gaps in the current knowledge of IAD pathogenesis addressed in this paper include: what is the role of ammonia? Does it just raise skin pH or does it have a more specific effect on the skin barrier function? Does the protease trypsin directly damage skin barrier function in a quantifiable way, both when applied to normal skin and when applied skin previously inoculated with urease expressing *Proteus mirablis*? The same question was asked with respect to the role of *C. albicans* and *S. aureus*, both believed to be involved in category 2 IAD: could they additionally damage skin barrier function following *P. mirablis*? Finally, could urease inhibition measurably prevent *P. mirablis* induced damage to skin barrier function? Integrity of skin models, consisting of in vivo human skin and ex vivo porcine skin, was monitored using impedance spectroscopy.^38^, ^39^

## 2 MATERIALS AND METHODS

### 2.1 General materials and instruments

Skin impedance was measured using a PalmSens4 potentiostat (PalmSens BV, The Netherlands). The stratum corneum moisture was measured using the MoistureMeterSC and trans‐epidermal water loss (TEWL) was measured using the VapoMeter (both from Delfin Technologies, Finland). Red Dot 3M Electrodes were sourced from Medisave, UK. Transparent film dressings (Hartmann, Germany), Aquacel Extra (Convatec, UK). Bacterial and fungal species were all freezer stocks or provided by colleagues at University of Bath: B4 *Proteus mirabilis,* H14320 *Proteus mirabilis, Staphylococcus aureus* and ATCC 60193 *Candida albicans*. Trypsin from bovine pancreas, urease from *Canavalia ensiformis* (Jack Bean), acetohydroxamic acid (AHA), components of artificial urine,^40^ broths, agars and ampicillin (Sigma‐Aldrich, UK). The porcine skin was taken from Oxford Sandy and Black pigs which was provided from a farm specialising in high welfare pigs. Pigs were slaughtered and butchered in a specialist abattoir, with particular care to avoid damaging the stratum corneum (no steam scalding or other treatment).

## 3 METHODOLOGY

### 3.1 Impedance measurement parameters and data fitting

A 10 mV amplitude sinusoidal potential was applied to the skin in two electrode mode via the 3M ECG electrodes, with the applied electrical frequency swept from 50 kHz to 0.2 Hz with 10 points being measured per decade. Data was fitted to an R1(R2Q) equivalent circuit using the PSTrace 5.8 software, where R1 is electrode resistance, R2 is resistance ascribed to stratum corneum integrity and Q a constant phase element related to skin capacitance.^37^ All skin impedance was measured before treatment to obtain baseline resistance.

### 3.2 Preparation of porcine skin

Dorsal ex vivo porcine skin was stored at −20°C. Skin was thawed at 32°C and 50% relative humidity. The porcine hair was trimmed to 1.5 mm, washed in PBS and air dried for 15 min. The full thickness skin samples, still attached to the subcutaneous fat, were approximately 1 cm thick.

### 3.3 Preparation of bacterial and fungal cultures

Microbial cultures were grown from −80°C freezer stocks on agar for 18 h at 37°C; B4 *Proteus mirabilis* was grown on non‐swarming Luria‐Bertani (LB) agar, H14320 *Proteus mirabilis* on non‐swarming LB agar containing 200 μg/ml of ampicillin, H560 *Staphylococcus aureus* on Tryptic Soy (TS) agar and ATCC 60193 *Candida albicans* on Yeast Extract‐Peptone‐Dextrose (YPD) agar. Single colonies were inoculated into 10 ml of LB broth (B4), ampicillin LB broth (H14320), TSB broth (H560) or Brain Heart Infusion (BHI) (ATCC 60193). The bacterial cultures were incubated at 37°C and *Candida albicans* at 32°C for 18 h at 200 rpm in a shaking incubator to obtain a population density of ∼10^9^ colony forming units per mL (CFU/ml).

### 3.4 Treatments on skin

Five separate inter‐linked studies of change in skin barrier function were carried out (Table 1): study 1 looked at the effect of urease enzyme (in artificial urine) on ex vivo porcine skin; study 2 looked at the effect of ammonia on in vivo human skin; study 3 looked at the combined effect of urease‐expressing bacteria and faecal enzymes on ex vivo porcine skin; study 4 looked at other opportunistic microorganisms on ex vivo porcine skin; study 5 looked the effect of inhibiting urease on in vivo human skin.

**TABLE 1:** Description of experimental protocols used to model IAD, including skin model, parameters, exposure times and number of replicates.

Study	Description	Model	Parameters	Exposure time/h	Replicates
**1**	Effect of urease	ex vivo porcine	Artificial urine Proteus mirabilis B4 P. mirabilis (10^9^ CFU/mL) P. mirabilis H14320 urease mutant (10^9^ CFU/mL)	24	4 (biological)
**2**	Effect of ammonia (EP23006)	in vivo human	Artificial urine Urease from Canavalia ensiformis (9 mg/mL) NH_4_OH (0.400 M, pH 10.5) NaOH (0.032 M, pH 10.5)	3	5 (biological) 3 (technical)
**3**	Effect of faecal enzymes	ex vivo porcine	Treatment 1: Artificial urine P. mirabilis B4 (10^9^ CFU/mL) Treatment 2: Artificial urine Trypsin (100 mg/mL)	Treatment 1:4Treatment 2:18	4 (biological)
**4**	Effect of opportunistic pathogens	ex vivo porcine	Artificial urine P. mirabilis B4 (10^9^ CFU/mL) Staphylococcus aureus H560 (10^9^ CFU/mL) Candida albicans ATCC 60193 (10^9^ CFU/mL) P. mirabilis B4 + S. aureus H560 P. mirabilis B4 + C. albicans ATCC 60193	3	4 (biological)
**5**	Effect of urease inhibitor (EP22107)	in vivo human	Artificial urine P. mirabilis B4 (10^9^ CFU/mL) P. mirabilis B4+ AHA (5 mM)	4	3 (biological) 3 (technical)

Abbreviations: AHA, acetohydroxamic acid; CFU/ml, colony forming units per mL.

For in vivo human studies, five volunteer participants, all University of Bath researchers aged 22–52, who gave informed consent were used for studies. All participants had a minimum of 1 week between different measurements to allow for skin recovery. Participants had skin sites selected from the dorsal upper arms and forearms. Each skin site measured 4 × 2 cm, allowing three measurements with ca. 1 cm diameter electrodes (technical replicates) per site. Up to six skin sites were used: upper dorsal forearm (one per arm); lower dorsal forearm (two per arm) and skin sites were a minimum of 4 cm apart. Measurement order was randomised to ensure that (for example) the upper dorsal forearm was not always measured first.

Variability in skin impedance across measurement sites and between participants was measured. In terms of variability across measurement sites, the median resistance values and interquartile ranges were evenly distributed across the anatomical regions. Therefore, the differences within individuals are likely to just be caused by natural fluctuations in thickness and hydration of the stratum corneum (Figure S4). However, a wide variation in skin resistance between different volunteers was measured by as much as three orders of magnitude (Figure S3). Hence the change in resistance was measured for each individual before and after treatment, and population group means were not used.

Skin was wiped with distilled water and dried. After 5 min, baseline measurements of skin barrier function were taken: skin impedance, trans‐epidermal water loss (TEWL), stratum corneum moisture and pH. Artificial urine was prepared according to Milo and Heylen *et al.* ^40^ For each replicate, 2.5 cm by 5 cm strips of Aquacel Extra (Convatec, UK) were inoculated with 3 ml of solutions containing artificial urine, in (Table 1). Once placed on skin, Aquacel was secured using transparent film dressing (studies 1–5), as well as a disposable nappy on top, to simulate higher temperature of skin under a nappy/pad (study 5).

### 3.5 Interpretation of skin impedance measurement

A previous study by the authors looked in detail at the physical origin of the resistive component of the measured skin impedance by a series of tape stripping experiments. These measurements showed that the measured resistance is primarily derived from the skin's stratum corneum.^38^

### 3.6 Statistical analysis

The data was plotted using GraphPad Prism 10, error bars depict the standard deviation of four biological replicates (ex vivo porcine skin) or three technical replicates (in vivo human skin) for each human participant. Statistical significance was assessed using an ordinary one‐way ANOVA. As discussed above, large population variation in skin resistance between individuals in the in vivo human studies meant that group means could not be used, with at least three biological replicates (three volunteers) were used for each study. The One‐Way ANOVA derived *p*‐value was used to tabulate significance of skin resistance change following treatment, allowing comparison of effects between different volunteers with very different baseline skin resistances (Table 2).

**TABLE 2:** One‐Way ANOVA *p* values, analysed on GraphPad Prism 10, of five in vivo human participants 0 h after treatment, comparing “artificial urine” against “urease”, “NH_4_OH” and “NaOH”.

	Urease		NH_4_OH		NaOH	
Participant	pH ↑	Impedance ↓	pH ↑	Impedance ↓	pH ↑	Impedance ↓
1	<0.0001	0.0010	<0.0001	0.0009	0.0004	ns
2	<0.0001	0.0156	<0.0001	0.0046	0.0001	ns
3	<0.0001	0.0063	<0.0001	0.0013	0.009	ns
4	<0.0001	0.0149	<0.0001	0.0159	ns	ns
5	<0.0001	ns (0.068)	<0.0001	0.0297	<0.0001	ns

### 3.7 Statement on ethics

Each study involving human volunteers was approved by the Bath Research Ethics Approval Committee: Study 2 (EP23006) and Study 5 (EP22107). Inclusion criteria: aged 18 or over and able to give informed consent. Exclusion criteria: irritated/abrased skin, pre‐existing dermatological conditions at the measurement sites and inability to give informed consent.

## 4 RESULTS

Most strains of *P. mirabilis* express urease and commonly found in the faecal/gut microbiome.^26^ Exposing skin to *P. mirabilis* provides a suitable model to investigate urease‐mediated damage, believed to contribute to IAD.

### 4.1 Study 1: Effect of urease (ex vivo porcine skin)

**FIGURE 2:**
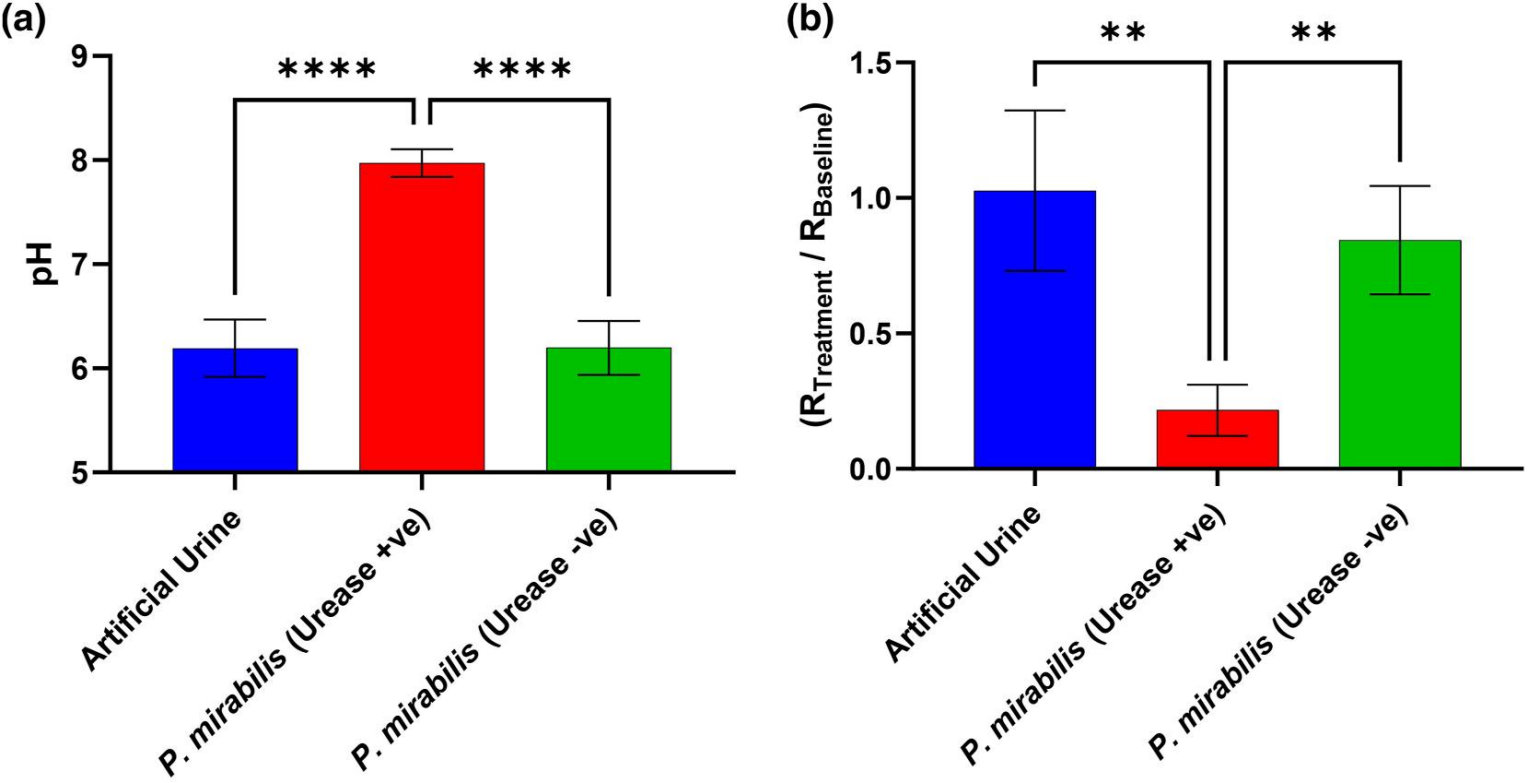
Skin pH (a) and resistance (b) of ex vivo porcine skin, subjected for 24 h to artificial urine with/out *Proteus mirabilis* strains B4 (urease‐positive) or H14320 (urease‐negative). Fitted resistance ‘R2’, from an R1(R2Q) circuit model, was normalised (R_Treatment_/R_Baseline_). Error bars represent the standard deviation of four independent replicates, analysed on GraphPad Prism 10 using an Ordinary One‐Way ANOVA: *p* ≤ 0.01 (**) and *p* ≤ 0.0001 (****).

The effect of urease on ex vivo porcine skin was studied in the absence of other enzymes/virulence factors by comparing a urease‐positive strain of *P. mirabilis* to a urease‐negative strain (Figure 2) after 24 h. As expected, the pH increased significantly in the presence of urease (Figure 1a), due to the conversion of urea within urine into ammonia. There was an apparent inverse correlation between pH and skin (stratum corneum) resistance (Figure 1b). The stratum corneum is composed of several layers of highly resistive dead cells; the reduction in resistance indicates that the skin barrier function was compromised by urease activity.^38^, ^41^ No trend was reported in the stratum corneum moisture and TEWL data (Figure S1), suggesting that the instruments were less sensitive to the experimental conditions compared to impedance spectroscopy. Further studies on *P. mirabilis* showed a time‐dependency for skin damage; 10^6^ CFU/ml causes significant damage after 8–10 h whilst 10^9^ CFU/ml requires 2 h (Figure S2).

### 4.2 Study 2: Effect of ammonia (in vivo human skin)

**FIGURE 3:**
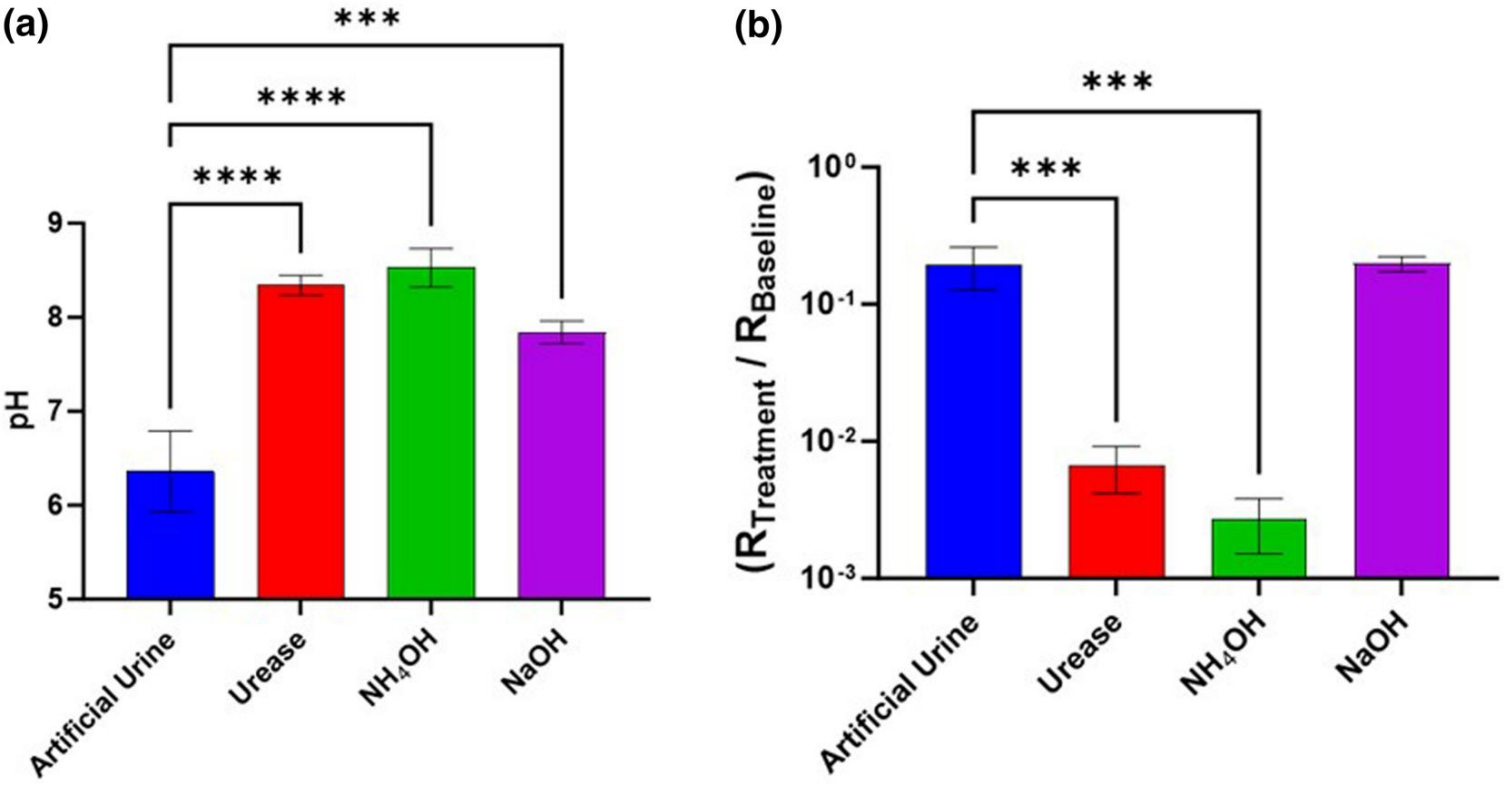
Skin pH (a) and resistance (b) of in vivo human (participant 1 of 5), subjected for 3 h to artificial urine (pH 6.1) with/out *Canavalia ensiformis*‐derived urease, NH_4_OH or NaOH (to achieve pH 10.5). Fitted resistance ‘R2’, from an R1(R2Q) circuit model, was normalised (R_Treatment_/R_Baseline_). Error bars represent the standard deviation of three independent replicates, analysed on GraphPad Prism 10 using an Ordinary One‐Way ANOVA: *p* ≤ 0.001 (***).

Whilst it is evident that urease activity causes skin damage (Figure 2), it is necessary to discern the mechanistic detail involved. As previously detailed, skin damage caused by elevated pH is well documented, but it is important to determine whether it is the key factor involved in IAD or if ammonia itself causes direct skin damage. This study was made on in vivo human skin, where exemplary data of Participant 1 is shown (Figure 3), involved exposure of five participants to solutions of artificial urine, at pH 6.1, or increased to pH 10.5 by addition of urease, NH_4_OH or NaOH. Pure urease (from *Canavalia ensiformis*) produces a similar effect to *P. mirabilis* (Figure S3), used here to achieve a faster rise in pH. Note, in water ammonia (NH_3_) reacts to form the ammonium hydroxide salt (NH_4_OH).

The effect of treatment on multiple participants is tabulated as whether statistically significant differences in skin resistance and pH were measured (Table 2). This provides a way to rapidly observe which conditions were causing changes in skin resistance/pH whilst controlling for the different baseline resistances (natural heterogeneity) of different participants' skin. Statistical significance is defined as *p* ≤ 0.05 from ONE‐WAY ANOVA tests.

Exposure of Participant 1 to the elevated pH solutions (urease, NH_4_OH, NaOH) overcame the skin's buffering capacity, causing a significant rise in skin pH (Figure 2a).^42^ This trend was consistent across all participants. Exposure to urease and NH_4_OH (in artificial urine) was consistently accompanied by a decrease in stratum corneum resistance which indicates skin damage (Figure 2b), although Participant 5 was marginally outside the range of significance, in terms of urease damage (*p* = 0.068). Crucially, NaOH did not result in a significant decrease in skin impedance across the five participants despite the increase in skin pH. Furthermore, exposure to NaOH did not cause skin erythema whilst urease and NH_4_OH did (Tables S1‐2). This suggests that ammonia itself causes direct irritation to skin, rather than the high pH. It is believed that skin inflammation is triggered by chronic exposure to urinary/faecal irritants, causing epidermal keratinocytes to release pro‐inflammatory cytokines.^4^, ^21^, ^22^ The specific irritants are not currently known but this study is important in showing that ammonia itself is the likely cause. This suggests that early treatment of IAD should focus on the specific damage caused by ammonia itself, either by inhibiting its production and/or sequestrating it by forming a non‐irritant adduct.^37^

Twenty‐four hours after the study, only Participant 2 retained a significant decrease in skin barrier function, following exposure to NH_4_OH (Table S3). This suggests that removal of the urease/ammonia irritant allows for recovery, the issue lies in prolonged exposure, as is the case for incontinent patients and nappy wearing children.

### 4.3 Study 3: Enhancement of activity of faecal enzymes by ammonia (ex vivo porcine skin)

**FIGURE 4:**
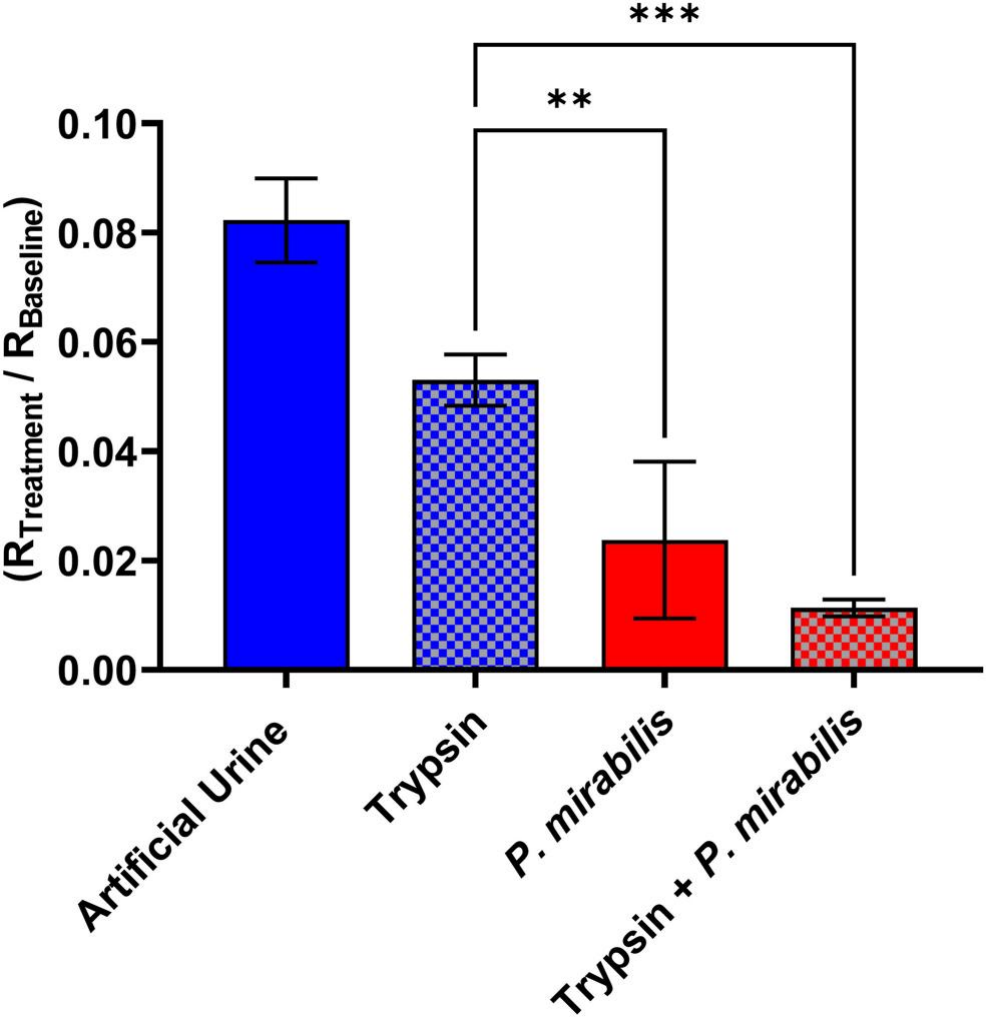
Skin resistance of ex vivo porcine skin, subjected for 4 h to artificial urine with/out B4 Proteus mirabilis, followed by 18 h of artificial urine with/out trypsin. Fitted resistance ‘R2’, from an R1(R2Q) circuit model, was normalised (R_Treatment_/R_Baseline_). Error bars represent the standard deviation of four independent replicates, analysed on GraphPad Prism 10 using an Ordinary One‐Way ANOVA: *p* ≤ 0.01 (**) and *p* ≤ 0.001 (***).

Trypsin is a key faecal enzyme which causes proteolytic damage to the skin (Figure 1). Studies showed that trypsin activity was increased in the following situations: elevated pH, including in the presence of urease (Figure S4 and S5), frictionally damaged skin (Figure S6) and in the presence of lipase (Figure S7). The capacity of urease to exacerbate trypsin‐related skin damage was investigated on ex vivo porcine skin (Figure 4). Whilst trypsin causes a significant decrease in resistance and corresponding skin integrity, the addition of urease‐positive *P. mirabilis* results in significantly greater damage than trypsin alone. Trypsin is known to play a role in digesting corneodesmosomes, loosening the intercellular connections between keratinocytes in the stratum corneum.^43^ Previous in vivo studies (Figure S5) showed that trypsin is more active in high pH solutions, including urease‐mediated alkalinity, meaning that skin digestion increases.^16^ Also, trypsin is an enhancement factor for transdermal penetration shown by Mugita *et al.* to facilitate deeper skin damage by intestinal bacteria.^6^ It is therefore likely that there is a mutually beneficial relationship (additive or synergistic) between trypsin and urease‐positive pathogens which explains the greater combined effect. There was no measured difference in stratum corneum moisture and TEWL, further suggesting that TEWL is less sensitive than impedance spectroscopy at measuring stratum corneum integrity, at least within the parameters of the IAD models used in this study (Figure S8).

### 4.4 Study 4: Enhancement of activity of opportunistic pathogens by ammonia (ex vivo porcine skin)

**FIGURE 5:**
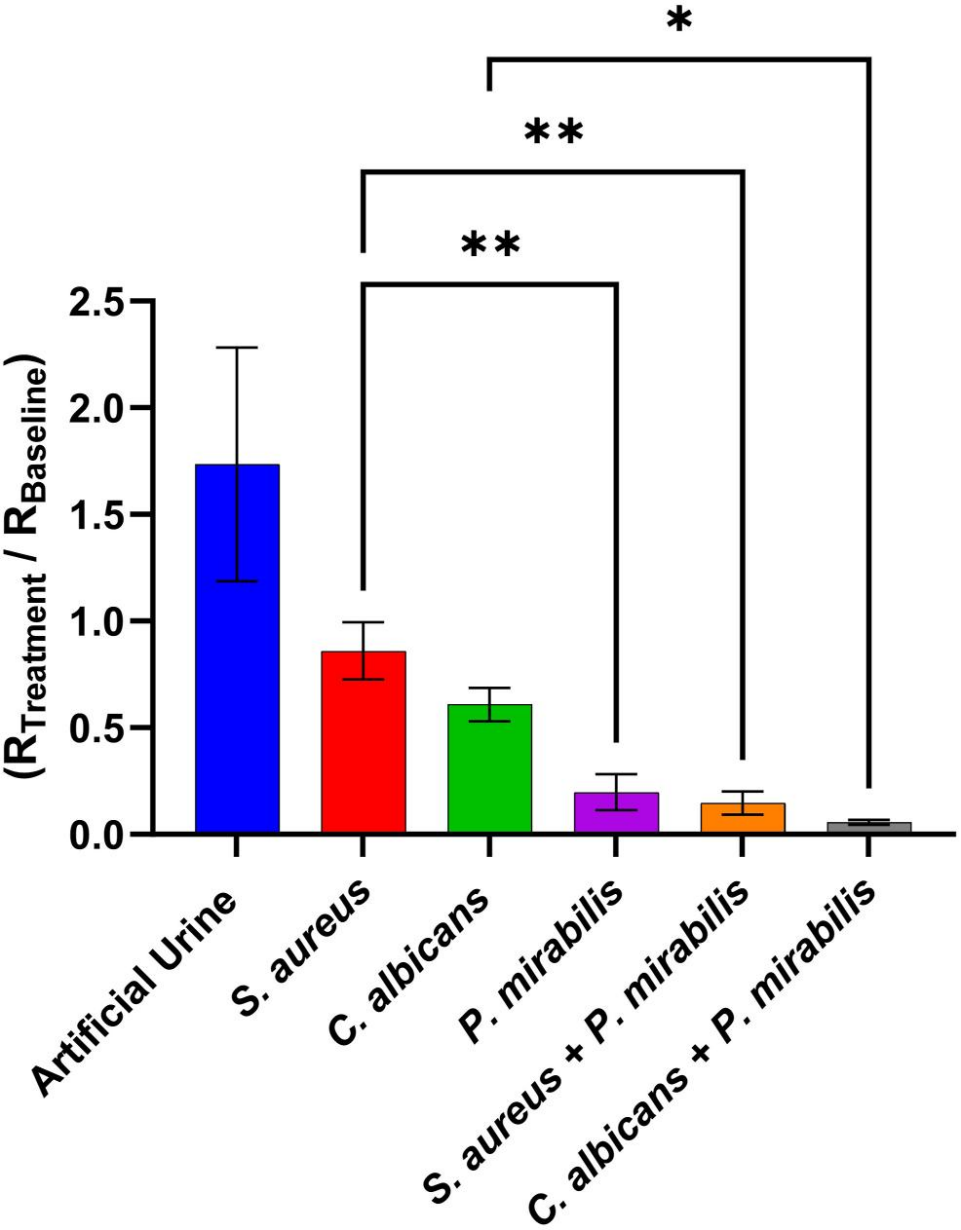
Skin resistance of ex vivo porcine skin, subjected for 3 h to artificial urine with/out H560 *Staphylococcus aureus*, ATCC 60193 *Candida* albicans and/or B4 *Proteus mirabilis*, followed by 18 h of artificial urine with/out trypsin. Fitted resistance ‘R2’, from an R1(R2Q) circuit model, was normalised (R_Treatment_/R_Baseline_). Error bars represent the standard deviation of four independent replicates, analysed on GraphPad Prism 10 using an Ordinary One‐Way ANOVA: *p* ≤ 0.05 (*) and *p* ≤ 0.01 (**).

The potential for a net additive/synergistic effect on skin damage by *P. mirabilis* and either *S. aureus* or *C. albicans* was explored (Figure 5). A high initial pathogenic loading of the respective pathogens (10^9^ CFU/ml) was used, to maintain consistency with previous work using *P. mirabilis* (Figures 2 and 4) and based on a 24‐h time point study where this loading was found to be most effective (Figure S9). After 3 h of skin inoculation, there was a significant reduction in skin resistance caused by each pathogen, compared to the negative control. Moreover, the measured skin resistance reduced significantly in the presence of *C. albicans* combined with *P. mirabilis*, compared to *C. albicans* or *P. mirabilis* alone. This suggests that the urease activity of *P. mirabilis* enhances the ability of *C. albicans* to damage the skin barrier, in support of the literature and our proposed model (Figure 1).^2^, ^19^, ^34^, ^35^ *C. albicans* is a polymorphic fungus; cells grow in ovoid‐shaped budding yeast at low pH, whilst at high pH (>7) hyphal growth is switched on. The hyphae are more invasive than the budding yeast form due to the ability to penetrate host cells and/or epithelial cell tight junctions.^44^ Whilst the same trend can be seen with *S. aureus*, this effect could not be separated from the activity of *P. mirabilis* alone, in the present study. Further work also showed an enhanced effect, in terms of skin damage caused by *C. albicans*, when in the presence of NH_4_OH (Figure S10).

### 4.5 Inhibition of urease

In the previous sections, urease was identified as a principal irritant responsible for direct and indirect skin damage. Incorporation of acetohydroxamic acid (AHA), a urease inhibitor, offers a therapeutic strategy by limiting ammonia production (Figure S11).

### 4.6 Study 5: Efficacy of AHA as a urease inhibitor in IAD management (in vivo human skin)

**FIGURE 6:**
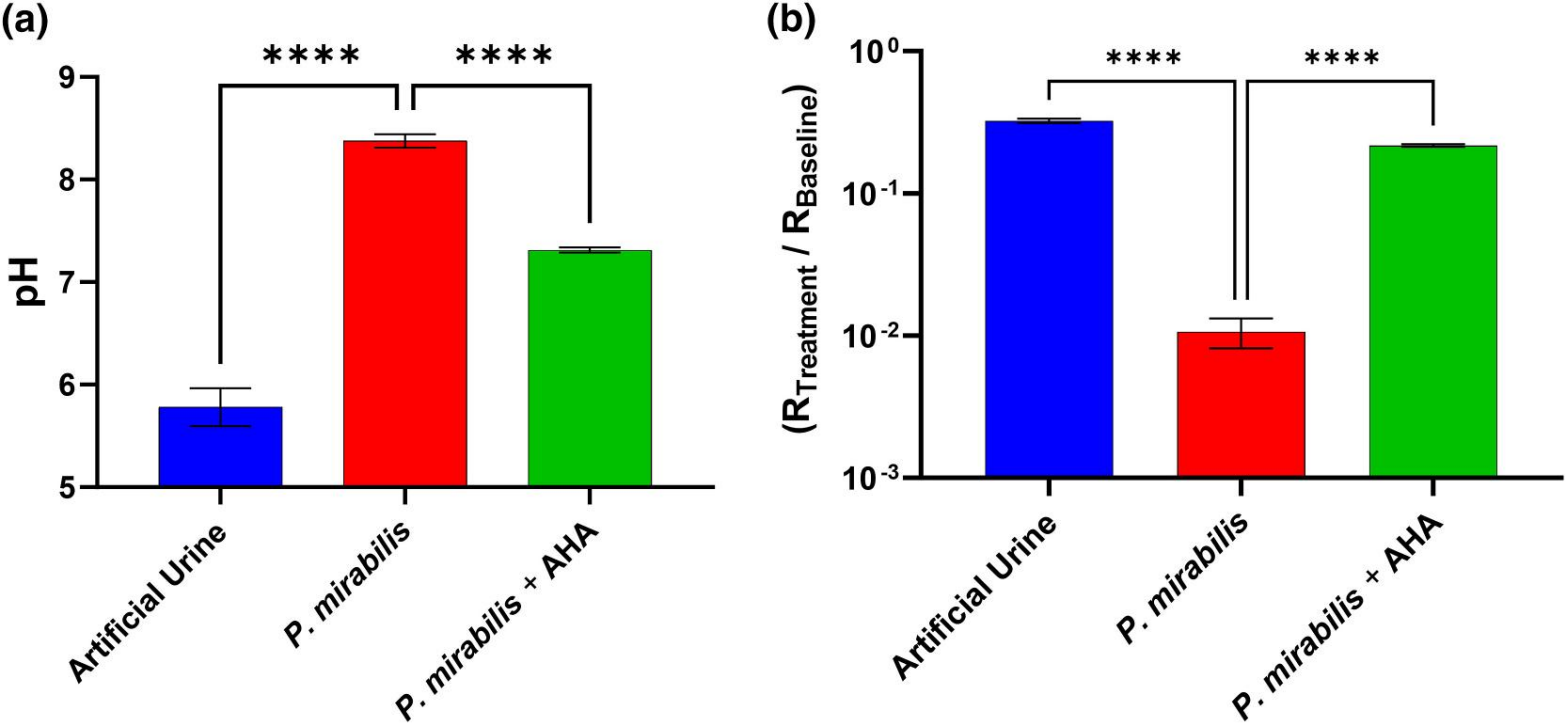
Skin pH (a) and resistance impedance (b) of in vivo human (replicate two on participant one of 3), subjected for 4 h to artificial urine with/out *Proteus mirabilis* B4 with/out acetohydroxamic acid (AHA). Fitted resistance ‘R2’, from an R1(R2Q) circuit model, was normalised (R_Treatment_/R_Baseline_). Error bars represent the standard deviation of three independent replicates, analysed on GraphPad Prism 10 using an Ordinary One‐Way ANOVA: *p* ≤ 0.001 (***).

Mugita *et al.* previously demonstrated the efficacy of urease inhibitors in preventing production of ammonia by *P. mirabilis* and subsequent increase in pH.^26^ However, it is also important to test this on an in vivo human model. The following study, where exemplary data of Participant 1 (Replicate 2) is shown (Figure 6), involved exposing three human participants to *P. mirabilis* and assessing the efficacy of AHA to minimise skin damage.

In terms of participant 1, the presence of AHA significantly minimised the increase in skin pH (Figure 6a), the decrease in resistance (Figure 6b) and prevented skin erythema (Table S4) associated with urease activity. These results were concurrent with preliminary studies on ex vivo porcine skin (Figure S12–S14). Assessment of the intra‐variability showed a consistent trend in replicate studies on Participant 1 (Table S5). In terms of participant inter‐variation, the trend was less consistent. Whilst Participants 1 and 2 displayed similar trends, AHA did not have a statistically significant effect on Participant 3. Considering that AHA does not completely prevent a rise in skin pH and therefore only partially inhibits urease under the experimental conditions used here, it is possible that participant 3 was more sensitive to urease/ammonia than participant 1 and 2.

## 5 DISCUSSION

Impedance Spectroscopy (multi frequency) has proved to be a valuable tool for measuring the damage to both in vivo and ex vivo skin from the micro‐organisms and enzymes involved in IAD. Comparing data from the ammonia versus sodium hydroxide study (Figure 3b) as well as the microbial and enzyme inoculation studies showed a clearly measurable decrease in skin resistance by greater than one order of magnitude. This compares well with Laser Doppler and Polarisation Spectroscopy (TiVi) and TEWL used by Larner et al to study the effect of ammonia applied to human volunteers' forearms in a study similar to this one, where the authors measured much smaller magnitude changes following ammonia addition to skin.^45^

The putative role of ammonia in IAD was reported 100 years ago.^46^ However recent reports of IAD pathogenesis often make less reference to ammonia and instead discuss observed elevation of skin pH in persons with IAD.^47^ This is important, as we demonstrate in this paper that it is ammonia (specifically the ammonium cation, NH_4_^+^) and not the hydroxide anion which causes skin damage. Current treatments for IAD are primarily focussed on removing the causative agent (urine/faeces), keeping skin clean and application of creams to the skin to create a physical barrier (although often with mildly antiseptic ingredients such as zinc oxide). No standard treatments currently target either the enzyme urease, or the ammonia produced by urease, although some topical ointments contain clotrimazole which inhibits *C. albicans*.

Topically applied urease inhibitors could potentially lower the virulence of urease‐positive pathogens and thus could be a future treatment strategy for IAD. As urease is a colonisation factor for many pathogenic microbes, inhibition could also cause a stable shift in the skin microbiome towards urease‐negative microbes. As a result, this could lead to the prevention of IAD and improve the skin microbiome and health. Different levels of microbial urease production can vary significantly between individuals, due to factors such as age and lifestyle, which may correlate with IAD manifestation.^48^, ^49^ Acetohydroxamic acid used in this paper is likely too toxic for general use, but urease is a potentially useful drug target for new small molecule inhibitory drugs with roles not just in the treatment of IAD but also other urease pathologies including urinary tract infection, bladder/kidney stones and GI tract infections.^50^ A further treatment possibility could be to actively sequestrate ammonia on the skin by molecules such as phenyl isothiocyanate which react with ammonia, forming the thiourea adduct.^51^ Phenyl isothiocyanate is found in various plant extract, including watercress. Hence watercress extract (or other isothiocyanate rich plant extracts) may have utility in future topical treatments for IAD.

*Limitations of the study:* The study has used models of IAD, including porcine skin and human skin, which both have limitations. Porcine skin is the closest animal skin model to human skin. The skin was removed from the pig (no scalding and hair carefully trimmed), then frozen and thawed once before use where structural integrity was likely reasonably preserved. However, the lack of vascularisation and immune response, due to being an ex vivo model, are considerable limitations. The in vivo human skin is a better model, however large variability in skin impedance between different persons was measured, although not between different measurement sites of the fore and upper arms of single volunteer subject. Arguably the principal limitation is that we were measuring healthy adult skin which is thicker and stronger than the skin of the two demographics who principally suffer from IAD: infants under 2 years of age and the elderly.

## 6 CONCLUSIONS

The enzyme urease, expressed by faecal pathogens, has two principal roles in the manifestation of IAD by catalysing the conversion of urea into ammonia. First, elevated skin pH which in turns activates faecal enzymes and opportunistic pathogens. Second, skin irritation by direct exposure to ammonia. This work suggests that urease is likely to be a key virulence factor which could be a potential pharmaceutical target in the development of therapeutics for the prevention and management of IAD. Such a strategy could reduce the irritation effect of prolonged exposure to urine/faeces, preventing manifestation of IAD and complications arising from opportunistic infections, including *C. albicans* and *S. aureus*.^2^, ^19^, ^34^

## CONFLICT OF INTEREST STATEMENT

Kyle Stewart is the co‐founder and CEO of Watercress research Ltd; Paul Winyard is the co‐founder and Chief Scientific Officer of Watercress research Ltd.

## AUTHOR CONTRIBUTIONS

**Emily Jane Owen**: Conceptualisation (equal); Formal analysis (equal); Investigation (lead); Writing – original draft (lead); Writing – review & editing (equal). **Rachel Heylen**: Conceptualisation (equal); Writing – review & editing (equal). **Kyle Stewart**: Conceptualisation (equal); Writing – review & editing (equal). **Paul Winyard**: Conceptualisation (equal); Writing – review & editing (equal). **Andrew Toby Jenkins**: Conceptualisation (equal); Formal analysis (equal); Funding acquisition (lead); Investigation (equal); Project administration (lead); Supervision (lead); Writing – review & editing (equal).

## ETHICS STATEMENT

Each study involving human volunteers was approved by the University of Bath Research Ethics Approval Committee: Study 2 (EP23006) and Study 5 (EP22107).

## Supporting information

Supplementary Material

## Data Availability

The data underlying this article is available in the supporting information or will be shared on reasonable request to the corresponding author.
