# Supplementary material for: The multi‐factorial modes of action of urease in the pathogenesis of incontinence associated dermatitis
Source: Skin Health Dis. 2024 Mar 2;4(3):e349. doi: 10.1002/ski2.349 (PMC11150740; doi:10.1002/ski2.349)
Supplement: Supplementary file 1 — Supplementary Material [file SKI2-4-e349-s001.docx]

**The Multi-factorial Modes of Action of Urease in the Pathogenesis of Incontinence Associated Dermatitis**

Emily J. Owen^1^, Rachel A Heylen^1^, Kyle Stewart^2^, Paul G. Winyard^2^, A. Toby A. Jenkins^1^

1. Department of Chemistry, University of Bath, Bath, BA2 7AY
2. Watercress Research Ltd. Unit 24, De Havilland Road, Skypark, Exeter, EX5 2GE

**Corresponding Author:** A. Toby A. Jenkins: [a.t.a.jenkins@bath.ac.uk](mailto:a.t.a.jenkins@bath.ac.uk)

| **A** | **B** |
| --- | --- |
| **** | **** |
| **C** | **D** |
|  |  |
|  | |

**Figure S1**. Skin pH (A), impedance (B), TEWL (C) and stratum corneum moisture (D) of *ex vivo* porcine skin, subjected for 6 h to artificial urine with/out B4 *P. mirabilis* (urease-positive), H14320 *P. mirabilis* (urease-negative) or NSM59 *E. coli* (urease-negative). Fitted resistance ‘R_2_’, from an R_1_(R_2_Q) circuit model, was normalised (R_Treatment_ / R_Baseline_). Error bars represent the standard deviation of four independent replicates, analysed on GraphPad Prism 10.

| **A** | **B** |
| --- | --- |
| 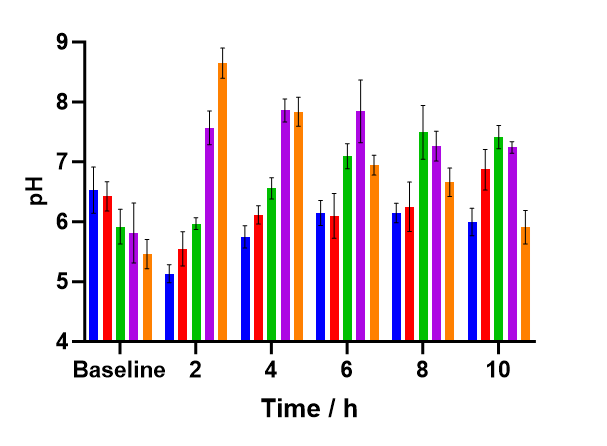 |  |
| **C** | **D** |
|  |  |
| 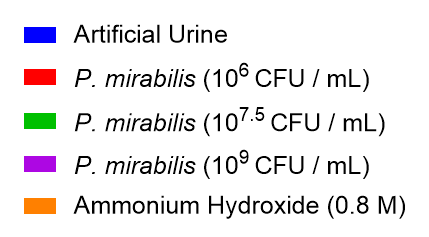 | |

**Figure S2.** Skin pH (A), impedance (B), TEWL (C) and stratum corneum moisture (D) of *ex vivo* porcine skin, subjected for 10 h to artificial urine with/out B4 Proteus mirabilis, at 10^6-9^ CFU / mL, or NH_4_OH. Fitted resistance ‘R_2_’, from an R_1_(R_2_Q) circuit model, was normalised (R_Treatment_ / R_Baseline_). Error bars represent the standard deviation of four independent replicates, analysed on GraphPad Prism 10.

|  |
| --- |

**Figure S3.** Skin pH impedance of *ex vivo* porcine skin, subjected for 24 h to artificial urine with/out *Canavalia ensiformis*-derived urease (0.09 ug / mL – 9 mg /mL). Fitted resistance ‘R_2_’, from an R_1_(R_2_Q) circuit model, was normalised (R_Time_ / R_Baseline_). Error bars represent the standard deviation of four independent replicates, analysed on GraphPad Prism 10.

**Table S1**. Erythema of five *in vivo* human participants, immediately after being subjected for 3 h to artificial urine (pH 6.1) with/out *Canavalia ensiformis*-derived urease, NH_4_OH or NaOH (to achieve pH 10.5).

| 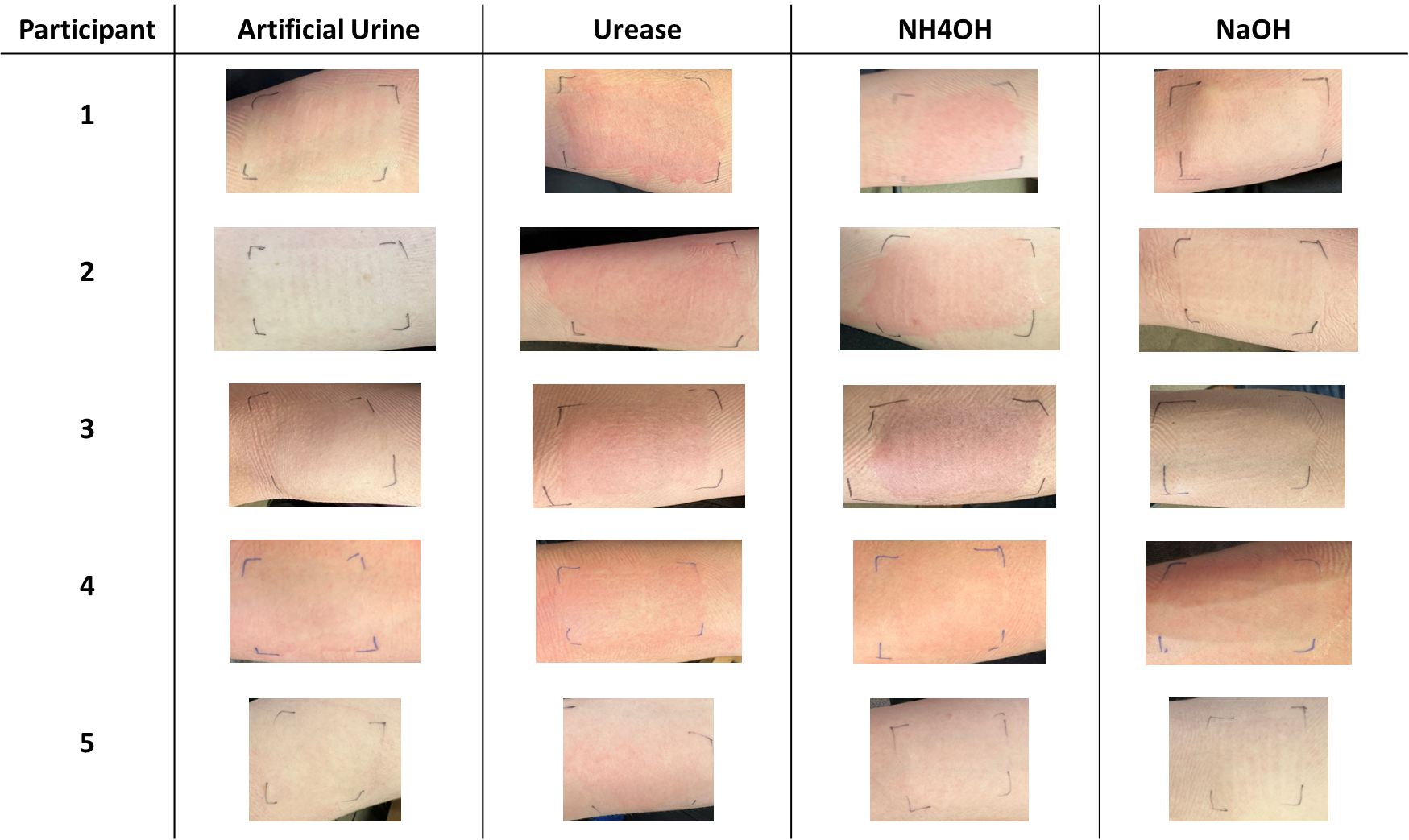 |
| --- |

**Table S2.** Erythema of five *in vivo* human participants, immediately after being subjected for 3 h to artificial urine (pH 6.1) with/out *Canavalia ensiformis*-derived urease, NH_4_OH or NaOH (to achieve pH 10.5).

| 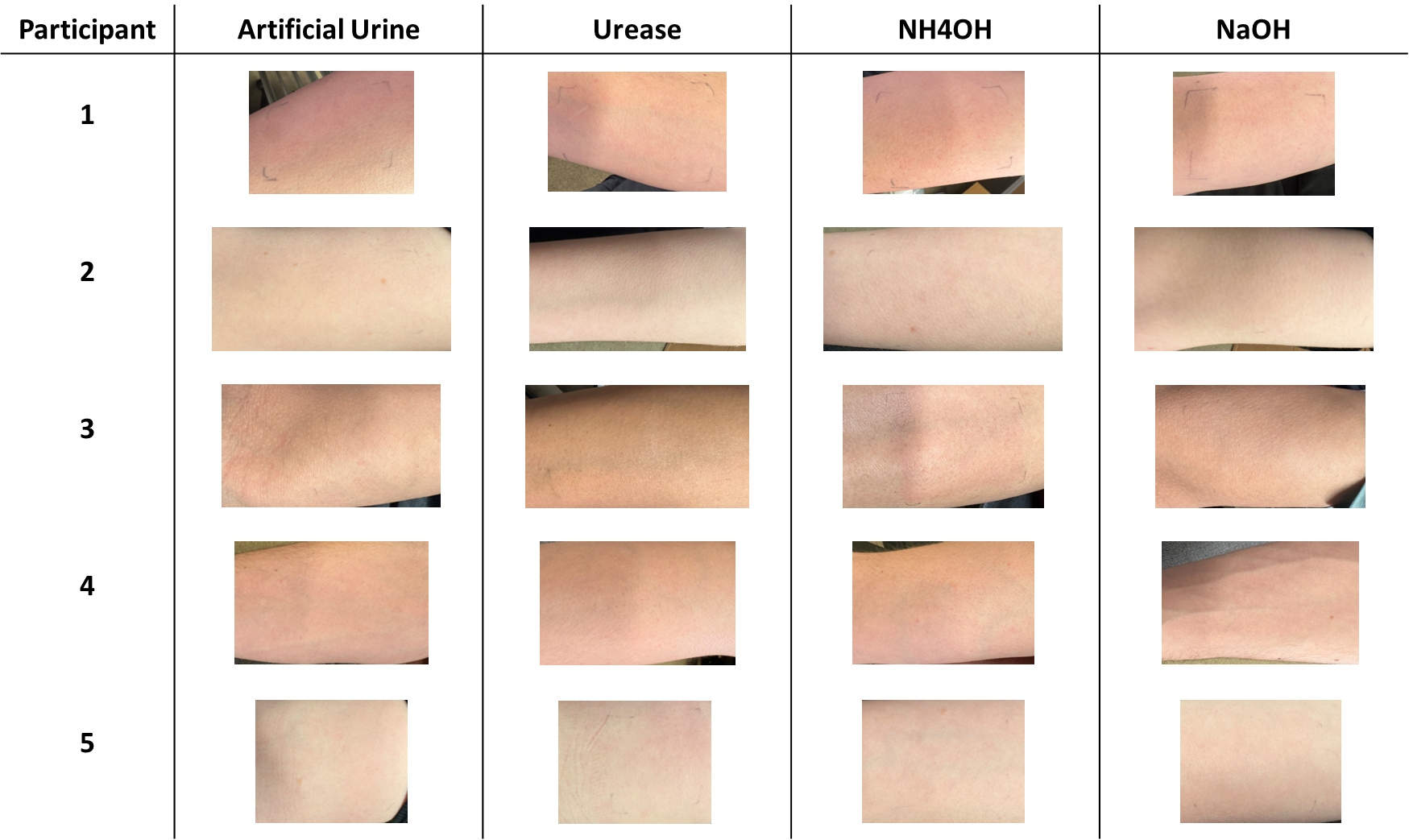 |
| --- |

***Inter-individual and Intra-individual Variation of measured skin impedance***

Skin impedance is predominately influenced by stratum corneum moisture and thickness, which can vary amongst individuals. The inter-individual and intra-individual variation in skin resistance was assessed in 22 healthy *in vivo* human participants by measuring their skin impedance in two proximal regions of each of their lateral upper arms, a lower site and an upper site (Figure S4).

The skin resistance varied by almost three orders of magnitude between the participants (Figure 3.17), ranging from 10^5^ to 10^8^ Ω. This demonstrates the importance of normalising data according to an individual’s baseline to separate inherent biological variation from experimental factors which change skin impedance over time. Furthermore, some individuals (particularly participant 1) displayed large intra-individual variation, despite proximal sites on the body being measured. Therefore, it is important that experimental conditions are kept constant by placing electrodes in the exact same regions of the body, in studies where repeat measurements of skin sites are taken over time.

| **** |
| --- |

**Figure S4** Skin resistance in 22 participants. Fitted resistance ‘R_2_’, was from an R_1_(R_2_Q) circuit model. Error bars represent the standard deviation of four independent replicates consisting of the lower/upper left/right lateral upper arms.

The same data was also plotted as a function of anatomical position: lower and upper regions of the left and right lateral upper arms (Figure S5). The reason for this was to determine whether there were consistent differences in skin impedance across the participants which could account for some of the intra-individual variation. Overall, the median values and interquartile ranges were evenly distributed across the anatomical regions. Therefore, the differences within individuals are likely to just be caused by natural fluctuations in thickness and hydration of the stratum corneum, as well as background interference with impedance measurements.

| **** |
| --- |

**Figure S5** Box plot of skin resistance values in 22 in vivo human participants in different regions of the lateral upper arms: LL is lower left, LR is lower right, UL is upper left, UR is upper right.

**Table S3**. ONE-WAY ANOVA p values of five *in vivo* human participants 24 hours after treatment, comparing “artificial urine” against “urease”, “NH_4_OH” and “NaOH”.

|  | **“Urease”** | | **“NH_4_OH”** | | **“NaOH”** | |
| --- | --- | --- | --- | --- | --- | --- |
| **Participant** | **pH ↑** | **Impedance ↓** | **pH ↑** | **Impedance ↓** | **pH ↑** | **Impedance ↓** |
| **1** | ns | ns | 0.0010 | ns | ns | ns |
| **2** | ns | ns | <0.0001 | 0.0100 | ns | ns |
| **3** | <0.0001 | ns | <0.0001 | ns | 0.0006 | ns |
| **4** | ns | ns | ns | ns | ns | ns |
| **5** | ns | ns | ns | ns | ns | ns |

|  |
| --- |

**Figure S6**. Standard curve of pNa absorbance at 430 nm. Error bars represent the standard deviation of four independent replicates, analysed on GraphPad Prism 10.

| **A** | **B** | **C** | **D** |
| --- | --- | --- | --- |
| **** | **** | **** | **** |
| **E** | **F** | **G** | **H** |
|  |  |  | 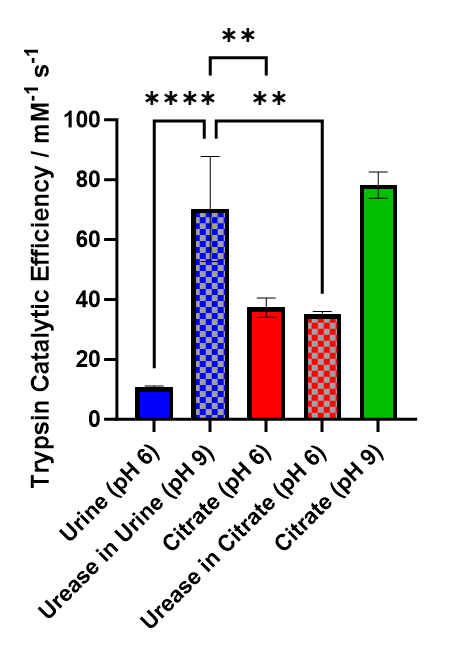 |

**Figure S7**. Trypsin activity in presence of BAPNA substrate, exposed to tris buffer (pH 6 to 9), in terms of V_max_ (A), K_m_ (B), K_cat_ (C) and catalytic efficiency (D), or mixtures of artificial urine and citrate buffer with/out urease, in terms of V_max_ (E), K_m_ (F), K_cat_ (G) and catalytic efficiency (H). Error bars represent the standard deviation of four independent replicates, analysed on GraphPad Prism 10 using an Ordinary One-Way ANOVA: *p* ≤ 0.05 (*), *p* ≤ 0.01 (**), *p* ≤ 0.001 (***) and *p* ≤ 0.0001 (****).

| **A** | **B** |
| --- | --- |
| **** | **** |
| **C** | **D** |
| **** | **** |

**Figure S8.** Skin pH (A), impedance (B), TEWL (C) and stratum corneum moisture (D) of *ex vivo* porcine skin, tape-stripped and subjected for 4 h to artificial urine with/out trypsin and/or *Canavalia ensiformis*-derived urease. Fitted resistance ‘R_2_’, from an R_1_(R_2_Q) circuit model, was normalised (R_Treatment_ / R_Baseline_). Error bars represent the standard deviation of four independent replicates, analysed on GraphPad Prism 10, using an Ordinary One-Way ANOVA: *p* ≤ 0.05 (*), *p* ≤ 0.01 (**), *p* ≤ 0.001 (***) and *p* ≤ 0.0001 (****).

|  |
| --- |

**Figure S9**. Skin impedance of *ex vivo* porcine skin, subjected for 17 h to artificial urine with/out lipase, followed by 8 h of artificial urine with/out trypsin. *Fitted resistance ‘R_2_’, from an R_1_(R_2_Q) circuit model, was normalised (R_Treatment_ / R_Baseline_). Error bars represent the standard deviation of four independent replicates, analysed on GraphPad Prism 10,* using an Ordinary One-Way ANOVA: *p* ≤ 0.05 (*) and *p* ≤ 0.01 (**).

| **A** | **B** |
| --- | --- |
| 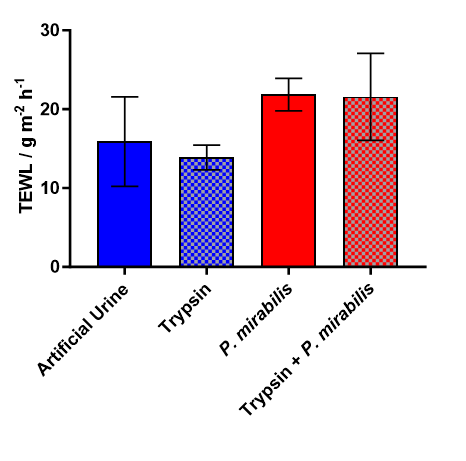 | 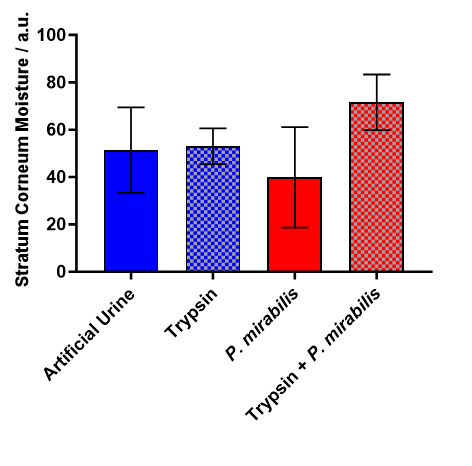 |

**Figure S10.** TEWL (A) and stratum corneum (B) of *ex vivo* porcine skin, subjected for 4 h to artificial urine with/out B4 *Proteus mirabilis*, followed by 18 h of artificial urine with/out trypsin. Error bars represent the standard deviation of four independent replicates, analysed on GraphPad Prism 10.

| **A** | **B** | **C** |
| --- | --- | --- |
|  | 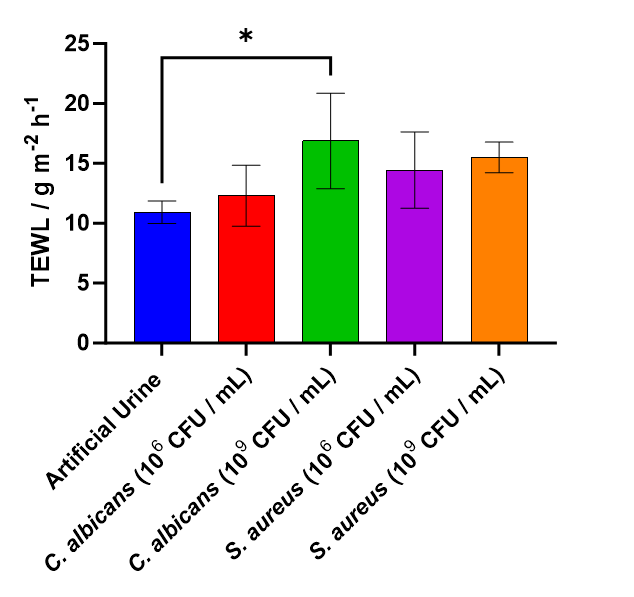 |  |

**Figure S11**. Skin impedance (A), TEWL (B) and stratum corneum moisture (C) of *ex vivo* porcine skin, subjected for 24 h to artificial urine with/out ATCC 60193 *C. albicans*, at 10^6-9^ CFU / mL, or H560 *S. aureus*, at 10^6-9^ CFU / mL. Fitted resistance ‘R_2_’, from an R_1_(R_2_Q) circuit model, was normalised (R_Treatment_ / R_Baseline_). Error bars represent the standard deviation of four independent replicates, analysed on GraphPad Prism 10, using an Ordinary One-Way ANOVA: *p* ≤ 0.05 (*) and *p* ≤ 0.01 (**).

| **A** | **B** | **C** |
| --- | --- | --- |
|  |  |  |
|  | | |

**Figure S12.** Skin impedance (A), TEWL (B) and stratum corneum moisture (C) of *ex vivo* porcine skin, subjected for 24 h to artificial urine with/out ATCC 60193 *C. albicans* and/or NH_4_OH. Fitted resistance ‘R_2_’, from an R_1_(R_2_Q) circuit model, was normalised (R_Treatment_ / R_Baseline_). Error bars represent the standard deviation of four independent replicates, analysed on GraphPad Prism 10.

|  |
| --- |

**Figure S13.** Change in pH of solutions of artificial urine with/out B4 *P. mirabilis* (10^6-9^ CFU / mL) with/out acetohydroxamic acid (AHA). Error bars represent the standard deviation of four independent replicates, analysed on GraphPad Prism 10.

| **A** | **B** |
| --- | --- |
| **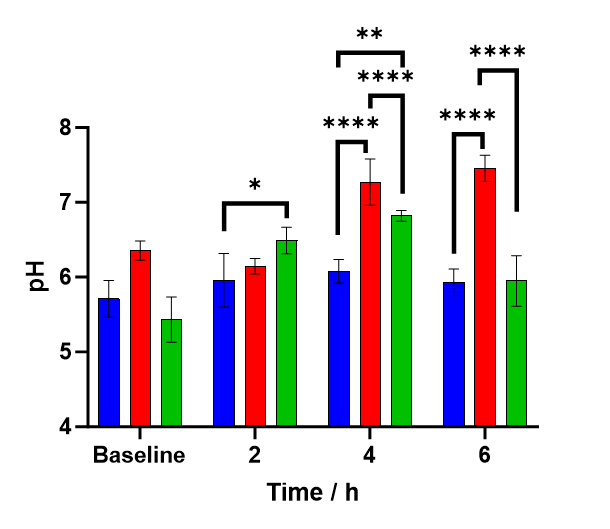** | **** |
| **C** | **D** |
| **** | **** |
| **** | |

**Figure S14**. Skin pH (A), impedance (B), TEWL (C) and stratum corneum moisture (D) of *ex vivo* porcine skin, exposed for 6 h to artificial urine with/out B4 *P. mirabilis* with/out acetohydroxamic acid (AHA). Fitted resistance ‘R_2_’, from an R_1_(R_2_Q) circuit model, was normalised (R_Treatment_ / R_Baseline_). Error bars represent the standard deviation of four independent replicates, analysed on GraphPad Prism 10, using an Ordinary One-Way ANOVA: *p* ≤ 0.01 (**) and *p* ≤ 0.0001 (****).

**Table S4**. Erythema of three *in vivo* human participants, immediately after being subjected for 4 h to artificial urine with/out B4 *Proteus mirabilis* with/out acetohydroxamic acid (AHA).

| 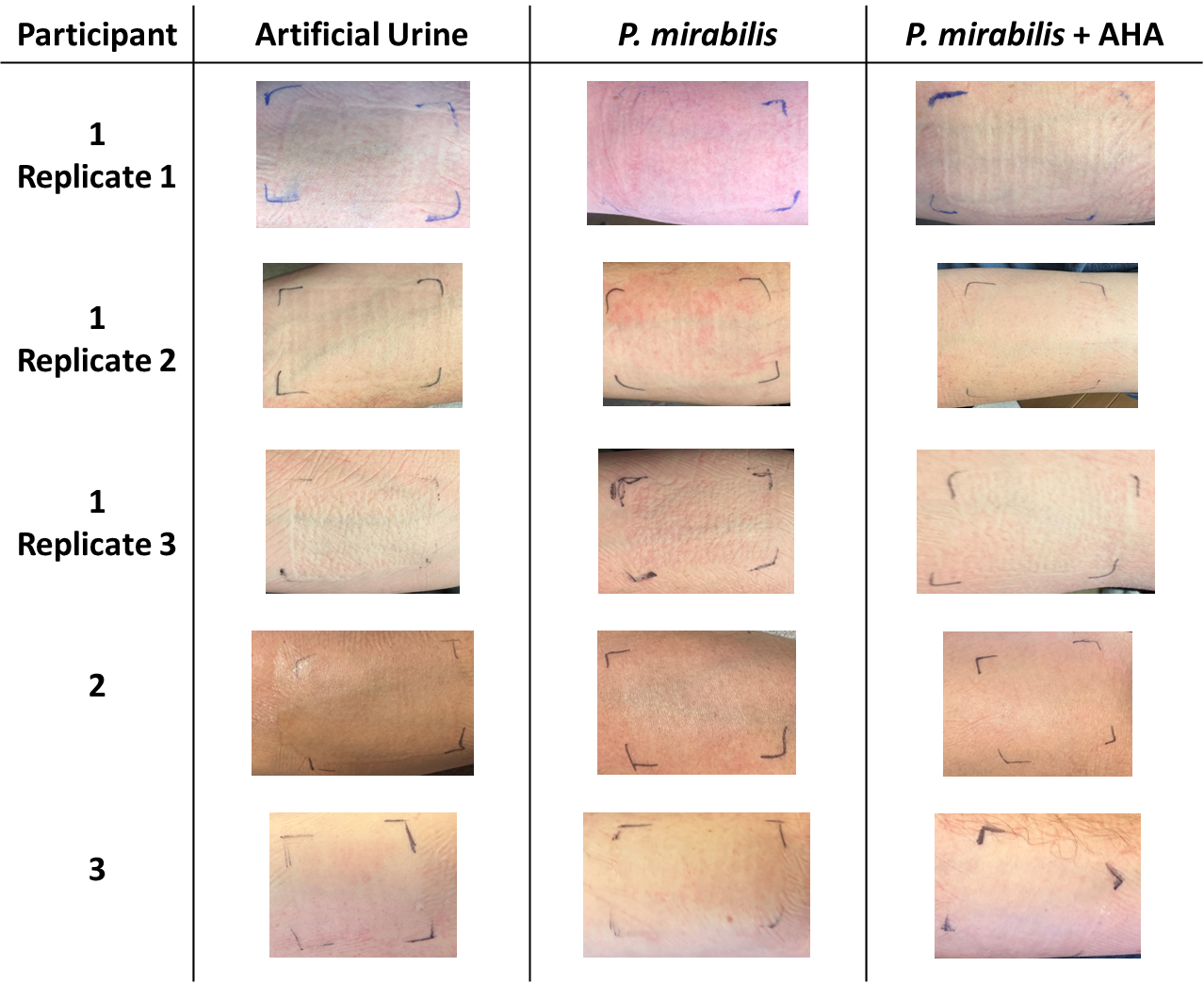 |
| --- |

**Table S5.** ONE-WAY ANOVA *p* values of the pH and impedance of three *in vivo* human participants after treatment, comparing “*P. mirabilis*” against “artificial urine” and “AHA”.

|  | **“Artificial Urine”** | | **“AHA”** | |
| --- | --- | --- | --- | --- |
| **Participant** | **pH ↓** | **Impedance ↑** | **pH ↓** | **Impedance ↑** |
| **1 (Replicate 1)** | <0.0001 | 0.0015 | 0.0001 | 0.0021 |
| **1 (Replicate 2)** | <0.0001 | <0.0001 | <0.0001 | <0.0001 |
| **1 (Replicate 3)** | <0.0001 | <0.0001 | 0.0020 | 0.0003 |
| **2** | <0.0001 | 0.0001 | 0.0003 | 0.0002 |
| **3** | <0.0001 | 0.0050 | 0.0007 | ns |

**Accuracy and parameters of the three principal biophysical analysers**

Not all manufacturers provide data on their instrument accuracy. A review of various non-invasive skin analysers is provided by Gidado et al: Iman M. Gidado, Meha Qassem, Iasonas F. Triantis, and Panicos A. Kyriacou, Review of Advances in the Measurement of Skin Hydration Based on Sensing of Optical and Electrical Tissue Properties, Sensors (Basel). 2022 Oct; 22(19): 7151. doi: 10.3390/s22197151

1. **Palmsens 4 instrument (see** [**https://www.palmsens.com/product/palmsens4/**](https://www.palmsens.com/product/palmsens4/) **)**

Potentiostat (controlled potential mode)

▪ applied dc-potential resolution 75 µV

▪ applied potential accuracy ≤ 0.1% ± 1 mV offset

▪ current ranges 100 pA to 10 mA (9 ranges)

▪ current accuracy ≤ 0.1 % at FSR1

▪ measured current resolution 0.006 % of current range (5 fA on 100 pA range

Impedance accuracy plot, as a function of applied frequency and measured impedance


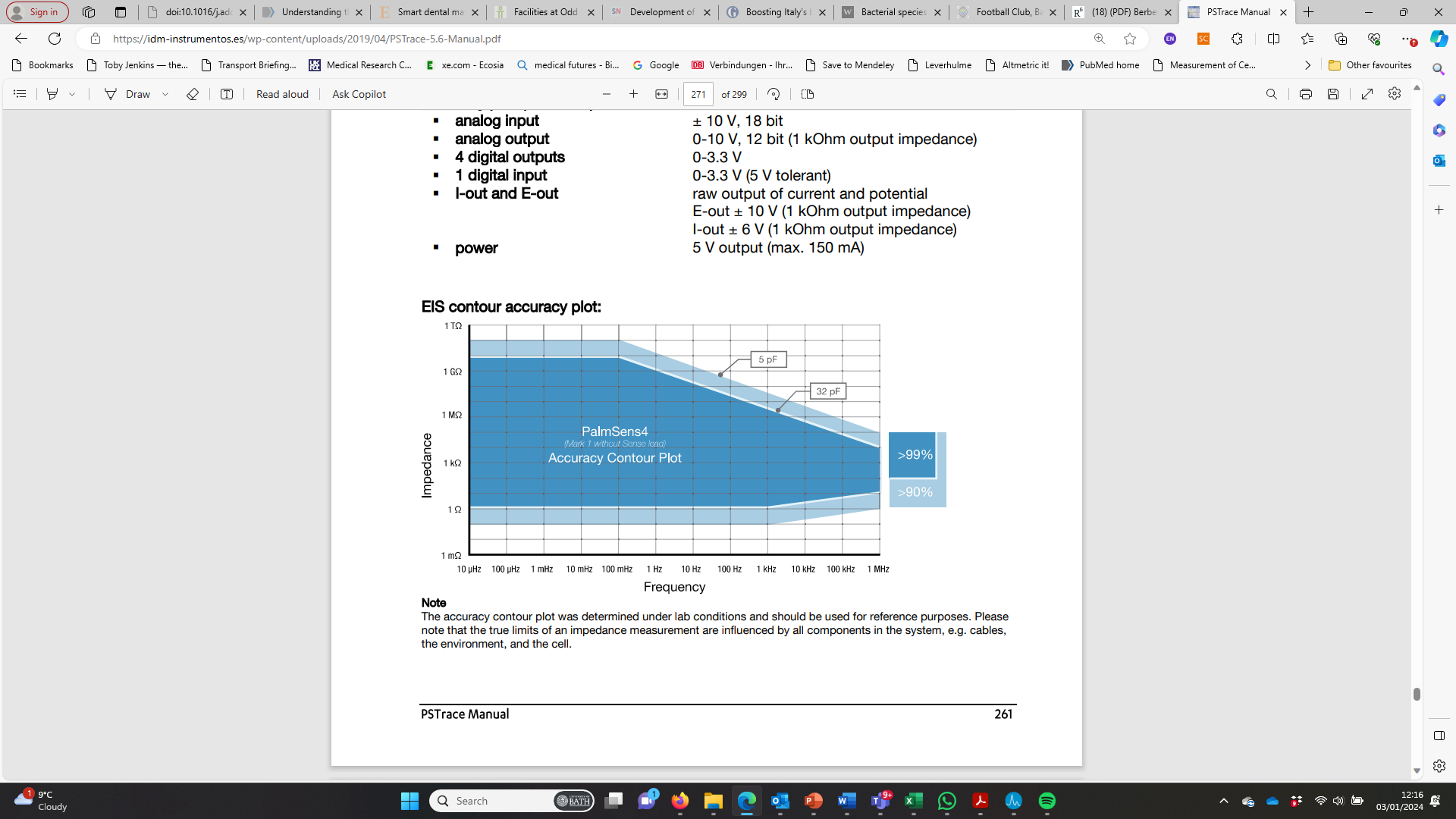


1. **Delfin Moisture Meter SC** <https://delfintech.com/products/moisturemetersc/>

This instrument makes a single frequency measurement at 1.25 MHz (much higher than the Palmsens 4 and interprets this in terms of skin moisture. Specific accuracy data is not provided by the manufacturers.

1. **Delfin Vapometer** <https://delfintech.com/products/vapometer/>

This instrument contains a humidity sensor mounted in a measurement chamber. The chamber is closed by the skin during the measurement period. The sensor monitors the increase of relative humidity (RH) inside the chamber during the measurement phase and the evaporation rate value (g/m²h) is automatically calculated from the RH increase. Specific accuracy data is not provided by the manufacturers.
